# Rebranding Nutritional Care for Critically Ill Patients

**DOI:** 10.2478/jccm-2023-0008

**Published:** 2023-02-08

**Authors:** Liliana Elena Mirea, Cristian Cobilinschi, Ioana Marina Grințescu

**Affiliations:** 1 Anaesthesia and Intensive Care Clinic, Clinical Emergency Hospital of Bucharest “Carol Davila” University of General Medicine, Bucharest, Romania

Since the organic and molecular roles and function of nutrients in supporting homeostasis for hospitalized patients have been already stated, remarkable advances have been achieved in the field of clinical nutrition [1]. Replacing the old terminology of “*nutritional support”* with the new concept of *“nutritional therapy”* both European Society for Clinical Nutrition and Metabolism (ESPEN) and American Society for Enteral and Parenteral Nutrition (ASPEN) emphasized that adequate nutrients administration reduces oxidative stress, metabolic response and sustains the immune response [1, 2]. The persistently increased prevalence of hospital malnutrition, inappropriate nutritional support during hospitalisation contributes undeniably to an increased mortality, especially in intensive care units [3].

In order to promote the importance of nutritional care and increase awareness among authorities and clinicians, “The International Declaration on the Human Right to Nutritional Care” was adopted during ESPEN Congress 2022 in Vienna. This Declaration highlights that nutritional therapy is a human right in the same manner as the right to food and health [4]. Moreover, all the undersigned societies, including Romanian Society of Enteral and Parenteral Nutrition (ROSPEN), raise awareness of the high prevalence of disease-related malnutrition along with the lack of access to appropriate nutritional support during and after hospitalisation.

Although sarcopenia was evaluated mostly in geriatric patients, is has been demonstrated that secondary sarcopenia associated with critical illness is an independent risk factor for prolonged hospital stay, weaning failure and mortality [5].

Considering the shifting metabolic status of critically ill patients which implies a variable energy expenditure, several tools were developed in order to better identify patients with highest nutritional risk[6]. Although scores such Nutrition-Risk 2002 (NRS-2002) or Nutrition Risk in Critically Ill score (NUTRIC) are commonly used worldwide, it is clear that these tools are not suitable for designing a personalised nutritional figure for intensive care (ICU) patients [7].

Besides the increased catabolism of proteins, in critically ill patients sarcopenia may also be promoted through sustained systemic inflammation, exacerbated oxidative stress, as well as through prolonged immobilization. While studies regarding acute loss of muscle mass in the ICU are already available, data concerning muscle mass status before ICU admission are mostly lacking [8].

As a result, current approach of nutritional therapy in ICU promotes body composition assessment, which should routinely go beyond body mass index calculation. Dynamic quantification and analysis of different compartments of the body proved to offer necessary data in order to tailor not only nutritional therapy, but also fluid therapy and medication, such as antimicrobial therapy [9]. Taking into account that only muscle mass was clearly linked to impact patients outcome, various techniques are now available for lean body mass quantification [10].

Anthropometric measurements are the first methods used for measuring body composition. Mid-arm or calf circumference are used for evaluating muscle mass, while triceps skin fold thickness reflects subcutaneous adipose tissue. Although these techniques may be old-fashioned, are still used today, because are inexpensive, non-invasive and easy to perform [11]. However, these methods are unreliable for critically ill patients, where rapid fluid shifts may occur.

Multifrequency bioelectrical impedance analysis (MF-BIA) may allow a reliable determination of body composition parameters, based on body water distribution. Although overhydration may be a limit for this monitoring technique, in order to obtain reliable data, it is recommended to be performed before fluid resuscitation (on admission) and after hydration shifts has been stabilized[9]. Recent studies have confirmed the applicability of MF-BIA in ICU, in which measured low phase angle was associated with severe outcomes [12]. Thibault et al demonstrated that bioelectrical impedance analysis-derived phase angle is an independent predictive factor for disease severity in ICU patients[13]. Convenient use of this cheap, rapid and easy technique may be a promising tool for body composition monitoring in intensive care settings [14].

Computed tomography (CT) is one of the most efficient monitoring techniques for body composition analysis, taking into account that it may provide reliable data regarding muscles cross sectional area and tissue’s density [14]. Jaitovich et al recently reported that skeletal muscle mass assessed through CT measurement of erector spinae muscle was significantly correlated with ICU survival and discharge [10]. Different measurements and various body areas were analysed using CT scanning, but psoas and paraspinal muscles linear measurements at the level of the third lumbar vertebrae were the most frequently reported [14, 15, 16]. Horibe et al indicated that muscle measurements obtained using this protocol and adjusting obtained data to age and sex, may be a reliable method to appreciate disease severity in patients with acute pancreatitis [15]. Nevertheless, it was suggested that cross-sectional muscle area measured at the level of the third lumbar vertebrae may be a good predictor of blood glucose variability [16]. But because CT scan is associated with increased radiation exposure and implies patients’ transportation, it is not feasible to use this imaging procedure solely for body composition assessment. However, it is well known that critically ill patients benefit for several CT scans during their ICU stay, thus a secondary retrospective evaluation of the results may usually be performed [17].

Magnetic resonance imaging (MRI) may provide accurate data regarding different body compartments [18]. Although is more advantageous than CT scan because it uses magnetic field gradients, MRI is not routinely used for body composition assessment in ICU, take into account higher costs, prolonged examination, and the necessity of MRI compatible equipment.

Dual-energy X-ray absorptiometry (DEXA) is currently the gold-standard technique for evaluation body composition, which is recommended for sarcopenia diagnosis by almost every international authority. A recent study conducted by Thackeray et al which evaluated lean body mass six months after ICU discharged, indicated that those who needed prolonged mechanically ventilation gained less lean mass and more fat mass, which was correlated with a poorer quality of life [19]. Besides the great enthusiasm surrounding this technique, it is worth mentioning that it’s use may be still limited considering increased variability of calibration, high costs and specific technical skills required.

Ultrasound has emerged as an essential tool for monitoring critically ill patients. This bedside method can also be used to assess muscle mass even in ICU patients with greater fluid shifts. Formenti et al reported that rectus femoris cross sectional area decreased to 10 % in the first seven days after ICU admission [20].

Another method frequently used for muscle mass assessment using ultrasound is by measuring quadriceps muscle layer thickness (QMLT), however in ICU patients in which overhydration may frequently occur QMLT may remain unchanged [21]. In a study in which were included critically ill mechanical ventilated patients was observed that QMLT measurement on day seven was an independent predictor for survival [22]. Taking into consideration the various advantages of using ultrasound for muscle mass assessment, more clinical data is expected, in order to establish diagnostic cut-off points [21].

Since lean body mass raises so much interest not only as a reflection of nutritional therapy, but also as an outcome parameter for critically ill patients, it is requisite that body composition measurement should routinely be performed.

Based on current evidence regarding body composition monitoring in critically ill patients, there is no technique that may be considered as gold-standard. Adopting a specific method should be based on staff experience, equipment availability and nevertheless, on patient’s course of the disease. Moreover, it should be considered that a sole result of an investigation is less valuable than a dynamic monitoring.
